# Systems biology approaches for the study of multiple sclerosis

**DOI:** 10.1111/j.1582-4934.2008.00375.x

**Published:** 2008-05-26

**Authors:** Francisco J Quintana, Mauricio F Farez, Howard L Weiner

**Affiliations:** Center for Neurologic Diseases, Harvard Medical SchoolBoston, MA, USA

**Keywords:** multiple sclerosis, systems biology, autoimmunity

## Abstract

Multiple sclerosis (MS) is a progressive neurological disease caused by an autoimmune attack to the central nervous system (CNS). MS is thought to result from a complex interaction between genetic and environmental factors. In this review, we analyse the contribution of genomics, trancriptomics and proteomics in delineating these factors, as well as their utility for the monitoring of disease progression, the identification of new targets for therapeutic intervention and the early detection of individuals at risk.

- Introduction
- Genomics
- Transcriptomics
  - Characterization of the MS lesion
  - Characterization of the immune response
- Proteomics
  - Identification of new pathogenic processes
  - Role of environmental triggers in MS
  - Characterization of the autoimmune response:
  - antibodies
- Metabolomics
- Integration of data from different ‘omics' approaches
- New experimental models
- Conclusions

## Introduction

Multiple sclerosis (MS) is an autoimmune disorder in which the central nervous system (CNS) is targeted by the dysregulated activity of the immune system, resulting in progressive neurological dysfunction. A variety of symptoms characterize MS, among them are visual and motor problems, changes in sensation in the arms, legs or face and weakness. At the onset of the disease, 85–90% of the patients present a clinical course characterized by discrete attacks followed by periods of partial or total recovery (relapsing-remitting MS, RRMS); 10% of the patients present a slowly accumulating disability over time (primary progressive MS, PPMS). A total of 40% of the patients initially diagnosed with RRMS eventually become progressive (secondary progressive MS, SPMS).

The term MS refers to the scars (scleroses or plaques) that characterize the white matter of the brain and spinal cord of MS patients. The autoimmune attack that drives MS is thought to cause these scars, characterized by a perivascular infiltration by inflammatory cells (B and T lymphocytes among them) [1, 2]. In addition, demyelination, astrogliosis and axonal injury are also detected [1, 2]. Different mechanisms contribute to axonal damage, including the direct effects of pro-inflammatory cytokines, complement fixation, apoptosis, cell-mediated cytotoxicity and neurodegeneration [1, 2]. Pathological findings suggest that the relative contribution of each one of these processes in disease progression differs in each patient [3].

The autoimmune response in MS targets components of the myelin sheaths surrounding neuronal axons, interfering with the neurons' ability to conduct electrical signals and probably leading to their death. Several CNS proteins are targeted by the immune system in MS, among them are myelin oligodendrocyte glycoprotein [4], oligodendrocyte-specific protein [5], myelin basic protein [6], myelin-associated glycoprotein [7], 2’,3’-cyclic nucleotide 3**’** phos-phodiesterase [8] and αβ-crystallin [9]. It is believed that the clinical symptoms that characterize MS result from the blockade in axonal transmission that follows axonal demyelination or axonal loss [1, 2].

Epidemiological studies have suggested the contribution of several environmental factors to the susceptibility of a specific individual to MS. Several viral, bacterial and parasitic infections have been classically linked to MS onset and progression [10–15], but no single environmental agent can be singled out as a ‘cause’ of MS.

MS is heterogeneous in its rate of progression, clinical symptoms, the specificity of the immune response and the pathology of the CNS lesions, reflecting the contribution of different factors to a pathogenic autoimmune response [16]. In this review, we analyse the contribution of genomics, trancriptomics, proteomics and metabolomics in delineating these factors, as well as their utility for the monitoring of disease progression, the identification of new targets for therapeutic intervention and the early detection of individuals at risk.

## Genomics

MS is considered a complex genetic disease in which many polymorphic genes have small effects on the overall disease risk, its severity, rate of progression and age of onset among several clinical outcomes. To date, the strongest chromosomal region linked to MS is the major histocompatiblity complex (MHC) locus on chromosome 6p21 [17–19]. In addition, several non-MHC candidate loci have also been linked to MS [19], but it has proven difficult to validate their association in independent studies. The difficulty in the identification of non-MHC genes associated to MS might reflect the genetic heterogeneity existing among MS patients, which results in different combinations of gene alleles leading to the same end phenotype. Nevertheless, polymorphisms in the α chain of the IL-7 receptor (IL-7Rα) have been recently associated with MS [20–22]. These polymorphisms make only a small contribution to the genetic susceptibility to MS but are a significant step towards the identification of genetic determinants for MS outside the MHC locus. The IL-7Rα allele associated with MS favours a relative decrease in the membrane-bound IL-7Rα[21]. IL-7 is produced by stromal cells in lymphoid tissues, its availability is controlled through its uptake by the membrane-bound IL-7R on T cells [23]. Thus, considering the positive effects that IL-7 has on lymphocyte survival and proliferation [23], the decrease in membrane IL-7R might result in increased levels of IL-7 available to fuel the inflammatory T-cell response in MS.

The α chain IL-2 receptor (IL-2Rα) gene has also been recently linked to MS [22]. IL-2Rα allelic variation has been previously associated to other autoimmune diseases such as type I diabetes, but at a different genomic position [24]. IL-2 is required for the development of regulatory T cells (T_reg_) [25, 26], and indeed, deficits in T_reg_ activity characterize RRMS [27]; thus the IL-2Rα polymorphisms might be related to the immune dysregulation observed in MS. Notably, IL-2Rα-specific antibodies have shown promising beneficial effects for the treatment of MS on phase 2 clinical trials [28, 29]. Although the link between IL-2Rα polymorphisms and MS is still awaiting further validation, the association of IL-7Rα and IL-2Rα variants to MS supports the use of genome-wide studies to delineate pathways contributing to disease pathogenesis.

## Transcriptomics

### Characterization of the MS lesion

Large-scale studies of mRNA expression have been directed at characterizing either the lesion or the immune response in MS. Lock and coworkers found that α4-integrin was found to be elevated in MS lesions [30]. α4-integrin mediates the interaction of T cells with the endothelium in the inflamed CNS, a required step for the migration of the self-reactive T cells into the brain and spinal cord in MS [31]. Antibodies to α4-integrin reverse and reduce the rate of relapse in relapsing-remitting experimental autoimmune encephalomyelitis (EAE) an animal model for MS [32], and a humanized version of this antibody showed positive effects in the treatment of RRMS [33].

In a separate study, the large-scale sequencing of non-normalized cDNA libraries derived from MS plaques revealed an increased expression of osteopontin (OPN) in the CNS of MS and EAE samples [34]. The up-regulation of OPN levels in MS plaques [35] and in the circulation [36–38] of MS patients was replicated in independent studies, prompting the search for polymorphisms in the *opn* gene associated with MS. Although some controversy still remains [39], polymorphisms in the opn locus have been associated with increased levels of circulating OPN and the clinical course of MS [40]. To study the mechanism of action of OPN in MS, OPN-deficient mice were generated, which showed a reduced severity in EAE [34]. OPN-triggered signalling is thought to contribute to MS pathogenesis by increasing the pro-inflammatory phenotype and survival of pathogenic myelin-specific T cells [41]. In addition, OPN interacts with the α4-integrin and is also involved in cell migration into the inflamed CNS [42]. Neutralization of OPN with neutralizing antibodies results in the amelioration of EAE [43]. Thus, OPN is therefore an example of how results obtained in transcriptomics studies might lead to the identification of mechanisms of disease pathogenesis and new therapeutic targets for MS.

### Characterization of the immune response

Transcriptional profiling has also been used to study the peripheral immune response in MS. Two limitations, however, should be kept in mind when using cDNA arrays for the analysis of the immune response in MS patients: First, these studies assume that changes in the peripheral immune system somehow reflect the immune response within the CNS. Second, the results of these studies are influenced by factors such as gender, age or changes in the relative proportion of different blood cell subsets that occur through the course of the disease. Nevertheless, two areas show significant progress in the transcriptional profiling of the immune response in MS: the follow-up of disease activity and the response to therapy.

Follow-up of disease activity: Achiron and coworkers characterized the transcriptional activity in peripheral blood mononuclear cells (PBMC) from RRMS patients during the course of the disease [44]. The authors identified a transcriptional signature associated to the relapse, that included genes involved in the recruitment of immune cells, epitope spreading and escape from immune-regulation. Although encouraging, these results should be validated using an independent set of samples and in longitudinal studies to assess their predictive value.

Response to therapy: β–Interferon (β–IFN) is widely used for the treatment of MS [45], however, biomarkers that would allow the identification of patients that would benefit from treatment with β–IFN are still not available. Weinstock-Guttman and colleagues used cDNA micro-arrays to study the effects of β–IFN therapy on monocyte-depleted PBMC [46]. They found significant changes in the expression of genes involved in the anti-viral response, β–IFN signalling and markers of lymphocyte activation. These studies provided a molecular description of the effects of β–IFN on RRMS patients and were later on extended to identify transcriptional signatures associated to a favourable response to treatment with β–IFN [47]. Based on these observations, Oksenberg and coworkers constructed a classifier for the identification of MS patients likely to respond to treatment with β–IFN [48]. The work of Oksenberg and coworkers is remarkable for two reasons: First, it demonstrates that gene expression profiling can be helpful in the selection of therapeutic regimes for the management of MS. Second, it uses a technology (real-time PCR) accessible to clinical laboratories, facilitating the translation of their results into daily medical practice.

## Proteomics

Proteomic studies in MS have been shown to identify new processes contributing to disease pathology and also, biomarkers for the early diagnosis and monitoring of MS patients.

### Identification of new pathogenic processes

Recently a large proteomic study of MS lesions by Steinman and coworkers have identified tissue factor and protein C inhibitor expression within chronic active plaque samples, suggesting that the dysregulation of the clotting cascade contributes to MS patho-genesis [49]. The authors went on to investigate the potential therapeutic use of their findings on EAE, concluding that the coagulation cascade is an attractive therapeutic target in MS.

### Role of environmental triggers in MS

Epidemiological studies suggest that environmental factors contribute to MS susceptibility. As a result, several groups are actively searching for microbial triggers for MS [10–15]. One of these putative triggers is the Epstein-Barr Virus (EBV) [50]. The link between EBV infection and MS has been recently strengthened by the work of Cepok and coworkers, who used protein expression arrays to characterize the antibody reactivity in the cerebrospinal fluid (CSF) of MS patients, most of those antibodies recognized EBV epitopes [51]. These results, together with the detection of EBV reactivation in active MS lesions [52], suggest that EBV might elicit an abnormal immune response in susceptible individuals that contributes to MS [53].

### Characterization of the autoimmune response: antibodies

The autoimmune nature of MS suggests that the study of the immune response should be useful for the early diagnosis, prognosis and monitoring of MS patients. T cells are thought to make a major contribution to MS immuno-pathology [16], but the standardized characterization of the T-cell response has proven difficult in MS. Antibodies might also have a pathological role [54]. Moreover, the activation of antibody-producing B cells is controlled by T cells, thus antibody response is thought to reflect the activity of the T-cell compartment [55]. Since it is easier to assay antibody reactivity than to follow antigen-specific T-cell responses, new technologies have been developed for monitoring the humoural response in MS patients and autoimmunity [56, 57].

Antigen arrays have been shown to detect changes in the repertoire of antibodies reflecting the antigen spreading that accompanies EAE progression [58]. The information obtained about the antigen spreading was used to design tailored immuno-modulatory vaccines to control EAE [58]. Of note, these vaccines showed promising results in a phase 1/2 human clinical trial [59].

Future experiments should study the antibody response in the serum of MS patients, searching for patterns of antibody reactivity that predict the progression of MS or the response to therapy, as it was shown for other autoimmune disorders, such as rheumatoid arthritis [60], autoimmune diabetes [57] and systemic lupus erythematosus [61]. Indeed, our own data suggest that antigen arrays might be used to identify antibody patterns linked to the different forms of MS and identify pathogenic mechanisms and therapeutic targets (F. J. Quintana *et al.*, submitted). Thus, antigen arrays are promising platforms for the identification of patients at risk of developing MS, before the overt onset of the symptoms [57].

## Metabolomics

The metabolome is defined as ‘the complete set of small-molecule metabolites (such as metabolic intermediates, hormones and other signalling molecules, and secondary metabolites) to be found within a biological sample, such as a single organism’[62]. Although initial studies aimed at studying the metabolome in simple organisms like the yeast [63], the study of a limited subset of the human metabolome in health and disease is well underway [64, 65]. Several groups have undertaken the study of metabolomic aspects of MS.

During the course of MS, macrophages and astrocytes produce nitric oxide, a metabolite that is thought to contribute to several aspects of MS pathology such as the disruption of the blood–brain barrier, oligodendrocyte injury and demyelination, axonal degeneration [66]. Nitric oxide metabolites can be detected in CSF, serum and urine of MS patients, and their levels seem to reflect the activity of inflammatory processes that contribute to the pathology of the disease [66–68].

N-acetylaspartate (NAA) is only present in living mature neurons, thus decreases in NAA levels indicate neuronal loss. Magnetic resonance (MR)-based approaches have been successfully used to measure the levels of NAA and other metabolites [69–72]*in vivo*, providing a novel non-invasive method for the acquisition of real-time data about the state of the CNS in MS. The power of this approach is highlighted by new data showing that the precise measurement of NAA levels by a combination of *in vivo* proton MR spectroscopic imaging with segmented, high-resolution MR imaging can identify RRMS patients in their transition to the SPMS form of the disease [73]. Although preliminary, these results suggest that metabolomics might provide sensitive biomarkers to follow up changes in the neurodegenerative and inflammatory processes that contribute to MS pathogenesis [74].

## Integration of data from different ‘omics' approaches

The combination of the data generated in transcriptomics and genomics studies can be an invaluable source of information and new hypotheses. Aune *et al.* compared the genes differentially expressed by lymphocytes in rheumatoid arthritis, systemic lupus erythematosus, insulin-dependent diabetes mellitus and MS, concluding that they are clustered within chromosomal domains in the genome [75]. Strikingly, they found that the chromosomal domains containing the genes differentially expressed in autoimmune disorders could be mapped to disease susceptibility loci associated to those diseases by genetic linkage studies [75]. These results suggest that the expression of disease-associated genes is co-regulated as a result of shared genetic regulatory elements or local patterns of chromatin condensation. Recently, Baranzini and coworkers studied the genetic concordance between gene expression and genetic linkage in MS [76]. They first compiled the data on gene expression available for MS and EAE, and superimposed it with all the known susceptibility loci identified in MS and EAE. In their study, Baranzini and coworkers identified the MS susceptibility genes located in the MHC locus as overlapping with clusters of differentially expressed genes in MS and murine EAE. However, they could also identify an interesting region on chromosome X that might contribute to the sexual dimorphism observed in MS. The integration of the data generated by different platforms, like transcriptomics, genomics and proteomics, is therefore likely to deepen our understanding of the mechanisms driving MS.

## New experimental models

Screenings aimed at identifying genes or drugs controlling the immune response cannot be easily undertaken in mice because they are based on crossing, maintaining and screening large numbers of animals, an expensive time-, space- and labour-intensive task; new experimental models are needed. Our current knowledge on innate immunity originated from pioneering studies that used flies and worms to carry out genetic studies and identify pathways controlling the response to microbes [77]. Invertebrates lack adaptive immunity, but the zebrafish (*Danio rerio*) harbours an adaptive immune system that resembles the mammalian immune system [78] and offers several experimental advantages for the study of pathways controlling vertebrate processes of interest. As part of our work on the zebrafish to identify pathways controlling immunity, we have characterized the zebrafish homologues of the transcription factors autoimmune regulator [79] and Foxp3 [80, 81], pivotal for central and peripheral tolerance, respectively.

Our work on zebrafish Foxp3 led us to identify the ligand-activated transcription factor aryl hydrocarbon receptor (AHR) as a regulator of the expression of mammalian Foxp3 [82]. Upon activation by its ligand 2,3,7,8-tetrachlorodibenzo-p-dioxin (TCDD), AHR interacts with its binding sites on the Foxp3 gene and stimulates its transcription. AHR activation by TCDD generates functional T_reg_ that inhibit the development of EAE by a transforming growth factor β1-dependent mechanism. Surprisingly, AHR activation by an alternative ligand, 6-formylindolo[3,2-b]carbazole, interferes with T_reg_ differentiation, boosts T_H_17 differentiation and worsens EAE. Thus, AHR regulates both T_reg_ and t_h_17 differentiation in a ligand-specific fashion, constituting a unique target for therapeutic immuno-modulation. In addition, our findings suggest that the experimental advantages offered by the zebrafish can be exploited to characterize metabolic pathways controlling immunity in vertebrates and to identify new targets for therapeutic intervention.

## Conclusions

How can we apply the information provided by genomics, transcriptomics and proteomics to the early diagnosis, prevention, monitoring and therapy of MS? A first step is the establishment of experimental models where biologic problems of interest can be investigated through genomic, transcriptomic and proteomic approaches simultaneously. The zebrafish, with its experimental advantages for the study of vertebrate-specific processes [78], might turn into a platform, where to identify pathways contributing to MS pathology and therapeutic targets. Our findings on the control of the immune response by AHR support this view.

Another step should be the adaptation of genomic, transcriptomic and proteomic technologies to a clinical setup. The personal genome project, for example, aims at developing fast and reliable methods to sequence the individual human genomes for US$1000 or less [83]. However, the data generated by these high-throughput approaches should be integrated to understand how the genomics, transcriptomics and proteomics of an individual influence each other. This would require the development of computational tools for the integration of networks and pathways into accurate quantitative models [84, 85]; with user interfaces aimed at facilitating its exploration and modification [86].

MS results from a complex dialogue between a susceptible individual and a fostering environment, a dialogue unique to each individual. The combination of genomic, transcriptomic and proteomic techniques might allow us to identify key elements in this dialogue to prevent, diagnose and cure MS.

## Abbreviations

2,3,7,8-tetrachlorodibenzo-p-dioxin (TCDD), aryl hydrocarbon receptor (AHR), β–Interferon (β–IFN), central nervous system (CNS), Cerebrospinal fluid (CSF), Epstein-Barr Virus (EBV), experimental autoimmune encephalomyelitis (EAE), IL-7 receptor α chain (IL-7Rα), IL-2 receptor α chain (IL-2Rα), magnetic resonance (MR), major histocompatiblity complex (MHC), multiple sclerosis (MS), N-acetylaspartate (NAA), nitric oxide (NO), osteopontin (OPN), peripheral blood mononuclear cells (PBMC), primary progressive MS (PPMS), regulatory T cells (T_reg_), relapsing-remitting MS (RRMS), secondary progressive MS (SPMS).
